# The Relationship Between Intolerance of Uncertainty, Sensory Sensitivities, and Anxiety in Autistic and Typically Developing Children

**DOI:** 10.1007/s10803-016-2721-9

**Published:** 2016-02-10

**Authors:** Louise Neil, Nora Choque Olsson, Elizabeth Pellicano

**Affiliations:** Centre for Research in Autism and Education (CRAE), UCL Institute of Education, University College London, 55-59 Gordon Square, London, WC1H 0NU UK; Center of Neurodevelopmental Disorders, Karolinska Institutet (KIND), Stockholm, Sweden; School of Psychology, University of Western Australia, Perth, Australia

**Keywords:** Sensory sensitivities, Anxiety, Intolerance of uncertainty

## Abstract

Guided by a recent theory that proposes fundamental differences in how autistic individuals deal with uncertainty, we investigated the extent to which the cognitive construct ‘intolerance of uncertainty’ and anxiety were related to parental reports of sensory sensitivities in 64 autistic and 85 typically developing children aged 6–14 years. Intolerance of uncertainty and anxiety explained approximately half the variance in autistic children’s sensory sensitivities, but only around a fifth of the variance in typical children’s sensory sensitivities. In children with autism only, intolerance of uncertainty remained a significant predictor of children’s sensory sensitivities once the effects of anxiety were adjusted for. Our results suggest intolerance of uncertainty is a relevant construct to sensory sensitivities in children with and without autism.

## Introduction

Autism spectrum disorders (ASDs) are most well known for the way they affect individuals’ social communication and interaction. Yet they are also characterised by non-social symptoms such as repetitive behaviours and activities and unusual responses to sensory input. These latter so-called ‘sensory sensitivities’ have risen to greater prominence with their inclusion in the recently revised diagnostic criteria for ASD, the DSM-5 (APA 2013). Such sensitivities are known to be common (Baranek et al. 2006; Tomchek and Dunn 2007), can cause much distress to individuals (Leekam et al. 2007), and have a negative impact on social relationships (Hilton et al. 2010) and family life (Baranek et al. 2006).

Sensory sensitivities also vary considerably between *and* within individuals on the autism spectrum (Grandin 2009; Hazen et al. 2014). They can include *hypo*-*sensitivity*, where an individual may seem to be unaware of, or slow to respond to, a stimulus that would normally be expected to elicit a response; *sensory seeking*, in which an individual exhibits an unusual behaviour such as a craving for, or intense interest in, certain sensory experiences; and *hyper*-*sensitivity*, where an individual is especially sensitive to sensory input. Autistic^1^ individuals can also experience *sensory overload*, where they become overwhelmed by incoming stimuli. In these situations, bright colours and lights, for example, can feel like a “bombardment” (Williams 1994, p. 22) and a hug like “a tidal wave of sensation” (Grandin 1992, p. 108).

Several accounts have been proposed to explain sensory sensitivities in autism, including weak central coherence (Frith and Happé 1994; Happé and Booth 2008), enhanced perceptual functioning (Mottron et al. 2006) and enhanced discrimination/reduced generalisation (Plaisted 2001). All of these accounts, however, have focused predominantly on superior processing at low levels, and thus fail to explain the full range of sensory sensitivities in autism.

More recently, computational accounts of sensory perception have attempted to explain sensory sensitivities in autism in terms of difficulties in generating expectations in regards to the sensory environment (Lawson et al. 2014; Sinha et al. 2014; Van de Cruys et al. 2014). Incoming sensory information is inherently noisy and ambiguous. Pellicano and Burr (2012) suggest that autistic individuals may have difficulties dealing with this ambiguity because they rely less on prior knowledge—due to difficulties either constructing internal working models of the world or combining them effectively with sensory signals—leading ultimately to a greater reliance on bottom-up sensory signals and a tendency to perceive the world more accurately or “as it really is” (p. 504). Without an internal template to guide interpretation, perceptual stimuli that should seem irrelevant may be enhanced, leading to sensory sensitivities such as hyper-sensitivity and sensory overload.

Difficulties dealing effectively with uncertainty are at the heart of Pellicano and Burr’s (2012) model and related accounts (Lawson et al. 2014; Van de Cruys et al. 2014), which all attempt to explain autistic perception at the computational and neural levels. Manifestations of perceptual differences in autism at the psychological level have hitherto been largely unexplored. One relevant construct, ‘intolerance of uncertainty’, characterised by the belief that uncertainty is negative and poorer functioning in situations of uncertainty (Buhr and Dugas 2002), has received much attention outside the autism literature, having been identified as a cognitive vulnerability factor for the development of generalised anxiety disorder (Carleton et al. 2012; Freeston et al. 1994) and also implicated in social anxiety (Whiting et al. 2014), obsessive compulsive disorder (Calleo et al. 2010) and depression (Carleton et al. 2012). The construct has typically been measured using a questionnaire (Carleton et al. 2007; Freeston et al. 1994), in which two factors have been identified: a *desire for predictability*, in which an individual perceives unexpected events as negative and craves sameness; and *uncertainty paralysis*, in which an individual is unable to act when faced with uncertainty (Birrell et al. 2011).

Two recent studies have investigated the relationship between intolerance of uncertainty and anxiety in autistic children. Boulter et al. (2014) found that increased levels of intolerance of uncertainty, as measured by the Intolerance of Uncertainty scale (Rodgers et al. 2012; Walker 2009), were associated with elevated levels of anxiety, as measured by the Spence Children’s Anxiety scale (Spence, 1998), in both children with (n = 114) and without autism (n = 110). In the same study, Boulter et al. showed that, once the effect of intolerance of uncertainty on anxiety had been taken into account, diagnostic group no longer significantly predicted anxiety, suggesting that high levels of intolerance of uncertainty might explain the high levels of anxiety consistently found in autistic children (Simonoff et al. 2008; White et al. 2009). Using the same questionnaire measures, Chamberlain et al. (2013) also reported increased levels of intolerance of uncertainty and anxiety in a group of autistic adolescents compared to a matched typically developing group, but failed to find any group difference in psychophysiological responses to an unpredictable threat (in the form of a puff of air to the neck).

Anxiety in autism has been repeatedly linked to individuals’ sensory sensitivities (Ben-Sasson et al. 2008; Green and Ben-Sasson 2010; Pfeiffer et al. 2005). Green et al. (2012) and Wigham et al. (2015) suggest one particular causal relationship: that sensory over-responsivity gives rise to anxiety. Yet there are other possible explanations of this link (Green and Ben-Sasson 2010). Following Pellicano and Burr (2012), difficulties dealing effectively with uncertainty at the computational or neural level may give rise to beliefs at the psychological level that uncertainty is negative and should be avoided. Desire to reduce this uncertainty, may lead to an increase in anxiety symptoms, such as ruminative thinking about various possible negative outcomes and hyper-vigilance to signs of threat in the environment. It is in these situations that individuals might be more likely to notice and react to aversive external sensory stimuli (Green and Ben-Sasson 2010). Only one study has investigated the association between intolerance of uncertainty and sensory sensitivities in autism. Wigham et al. (2015) reported a moderate positive correlation between scores on the Intolerance of Uncertainty scale and sensory over-responsiveness, as measured by the Short Sensory Profile in children with autism (n = 53).

The current study sought to extend the work of Wigham et al. (2015) by examining the relationship between intolerance of uncertainty, sensory sensitivities and anxiety in groups of children with and without autism. Our study had four aims. First, we examined between-group *and* within-group differences on all three variables. Like previous studies (Boulter et al. 2014; Tomchek and Dunn 2007), we expected that parents would report elevated levels of intolerance of uncertainty, sensory sensitivities and anxiety in their autistic children, relative to typical children, and that there would be strong positive links between these variables within each group.

Second, we tested the veracity of the aforementioned causal account regarding the relationship between intolerance of uncertainty, sensory sensitivities and anxiety by examining the role of anxiety as a potential mediating factor in the relationship between intolerance of uncertainty and sensory sensitivities.

Third, we examined potential differences, if any, in the nature of the relationships between these three constructs between autistic *and* typically developing children. While Boulter et al. (2014) reported a similarly sized significant and positive relationship between intolerance of uncertainty and anxiety in both autistic children and typical children, implying that intolerance of uncertainty plays an equally important role in the development of anxiety in both groups, it remains unknown whether the relationship between intolerance of uncertainty and sensory sensitivities follows a similar pattern in both autistic and typical children.

Finally, we extended Boulter et al.’s (2014) work, which found that intolerance of uncertainty mediated the relationship between autism diagnosis and anxiety, to determine whether intolerance of uncertainty might also explain the relationship between autism diagnosis and sensory sensitivities.

## Methods

### Participants

The primary caregivers of 64 autistic children aged from 6 to 14 years (55 boys; M = 10.36, SD = 2.36) and 85 typically developing children (43 boys; M = 9.15, SD = 1.87), took part in this study. Families were recruited through advertisements on the University and UK’s National Autistic Society webpages, the Autism Spectrum Database-UK (www.ASD-UK.com), mainstream and special schools and parent support groups in the Greater London area. Parents completed the Social Communication Questionnaire (SCQ; Rutter et al. 2003) and researchers administered the Autism Diagnostic Observation Schedule (ADOS-G or ADOS-2; Lord et al. 2000, 2012) to autistic children using the revised algorithm (Gotham et al. 2007, 2008). All children with autism scored above threshold for an ASD on one or both of these measures (see Table 1 for scores). Two additional children with autism were assessed but excluded for scoring below the cut-off on both the SCQ and the ADOS. Furthermore, all autistic and typically developing children obtained an IQ score of at least 70, as measured by the WASI-II (Wechsler 2011). All 149 primary caregivers participating in this study completed all three questionnaire measures.

**Table 1:** Group characteristics of autistic and typically developing children

Measures	Autistic children (n = 64)	Typically developing children (n = 85)
Age (years)		
M (SD)	10.36 (2.36)	9.15 (1.87)
Range	6.37–14.70	6.00–13.76
Verbal IQ		
Mean (SD)	97.33 (16.36)	111.02 (12.16)
Range	57–130	79–149
Performance IQ		
Mean (SD)	100.31 (15.40)	105.62 (13.21)
Range	73–134	75–132
Full-scale IQ		
Mean (SD)	98.58 (14.93)	109.47 (11.64)
Range	70–129	78–135
SCQ		
Mean (SD)	24.27 (9.01), n = 63	3.54 (3.24), n = 84
Range	5–46	0–14
ADOS		
Mean (SD)	10.42 (3.75), n = 55	
Range	3–20	

Verbal IQ, performance IQ and full-scale IQ were all measured using the WASI-II (Wechsler 2011)

SCQ = Social Communication Questionnaire (Rutter et al. 2003). A score of 15 or above indicates elevated levels of autistic symptomology; ADOS = Autism Diagnostic Observation Schedule (Lord et al. 2000, 2012). Scores of 7 or above indicate the presence of an ASD

### Measures

#### Intolerance of Uncertainty Scale: Parent Version

The Intolerance of Uncertainty scale is a 12-item questionnaire adapted for parents by Rodgers et al. (2012). Parents are asked to indicate the extent to which a list of statements is like their child, on a scale from 1 (not at all like them) to 5 (entirely like them). Scores across the 12 items are summed to yield a total score; higher scores reflect greater levels of intolerance of uncertainty. The scale is a modified version of the 27-item Intolerance of Uncertainty scale originally developed by Freeston et al. (1994) then shortened by Carleton et al. (2007) and adapted further by Walker (2009). The parent version of the Intolerance of Uncertainty scale has been found to have excellent internal consistency in both parents of children with autism (α = 0.90) and parents of typical children (α = 0.90; Boulter et al. 2014). In the current study, the scale also showed excellent internal consistency among all parents (children with autism: α = 0.90; typically developing children: α = 0.88).

#### Short Sensory Profile

The Short Sensory Profile is a 38-item adaptation of the Sensory Profile (Dunn 1999). The behaviours measured in the scale cover seven domains: tactile sensitivity, taste/smell sensitivity, movement sensitivity, under-responsivity/seeking sensation, auditory filtering, low energy/weakness and visual/auditory sensitivity. Parents rate the frequency of each item on a scale from 1 (always) to 5 (never). Scores are summed to yield a total score in which lower scores reflect higher levels of sensory sensitivities. McIntosh et al. (1999) demonstrated good psychometric properties for the scale, including adequate internal consistency of the total and subscale scores (Cronbach’s alpha ranged from 0.70 to 0.90), good convergent validity with physiological measures and a discriminant validity of >95 % in distinguishing children with and without sensory modulation difficulties. In the present study, the scale showed excellent internal consistency in both parents of autistic children (α = 0.93) and parents of typical children (α = 0.94).

#### Spence Children’s Anxiety Scale: Parent Version

The SCAS-P is a 38-item parent report measure of children’s anxiety (Nauta et al. 2004), adapted from the original child version of the scale (Spence 1997, 1998). The scale can be divided into six subscales in accordance with anxiety disorders outlined in the DSM-IV (APA 1994) including generalised anxiety, panic and agoraphobia, social phobia, separation anxiety, OCD and specific fears or phobias (labelled by Spence as ‘physical injury fears’). Respondents rate the frequency of each item on a 4-point Likert scale ranging from 0 (never) to 3 (always). Scores across all 38 items are summed to yield a total score; higher scores reflect greater levels of anxiety. Nauta et al. (2004) reported good to excellent internal consistency in the scale (Cronbach’s alpha of 0.89), as well as good convergent and discriminatory validity and support for its six-factor structure. It has also demonstrated good psychometric properties as a measure of anxiety among children and adolescents with autism (Zainal et al. 2014). In the current sample, the scale showed excellent internal consistency in the parents of both autistic (α = 0.93) and typically developing (α = 0.87) children.

### Procedure

This study was part of a larger investigation into sensory sensitivities in autistic children. Children were seen individually in one or more face-to-face sessions at the university during which they were administered the Wechsler Abbreviated Scales of Intelligence, Second Edition (WASI-II; Wechsler 2011), as a measure of intellectual functioning and, for autistic children (n = 55), the Autism Diagnostic Observation Schedule—Second Edition (ADOS-2; Lord et al. 2000, 2012). Parents were asked to complete the Social Communication Questionnaire, Short Sensory Profile, Spence Children’s Anxiety scale and Intolerance of Uncertainty scale.

This study was granted ethical approval by the University’s Research Ethics Committee. Written informed consent was obtained from all parents prior to their and their child’s participation.

## Results

### Between-Groups Analysis

To begin, and to address our initial aim, we examined group differences in scores on the Intolerance of Uncertainty scale, Short Sensory Profile and the Spence Children’s Anxiety scale. Table 2 shows scores on each of the measures for autistic children (n = 64) and typical children (n = 85). Although typically developing children’s scores on both the Short Sensory Profile and Intolerance of Uncertainty scale showed a slight positive skew, and autistic children’s a slight negative skew, all scores followed a relatively normal distribution in both groups. Log and square root transformations did not improve the distribution of scores, so data were left untransformed.

**Table 2:** Descriptive statistics by group for total scores on the Intolerance of Uncertainty scale, Short Sensory Profile and Spence Children’s Anxiety scale in children with autism and typically developing children

Measures	All autistic children (n = 64)	All typically developing children (n = 85)	Matched autistic children (n = 40)	Matched typically developing children (n = 40)
IUS				
Mean (SD)	39.70 (11.19)	21.02 (8.39)	38.93 (10.87)	20.83 (8.72)
Range	18–59	12–47	18–59	12–42
SSP				
Mean (SD)	114.78 (28.34)	166.56 (18.77)	113.08 (28.18)	166.45 (20.86)
Range	63–181	96–190	63–181	96–190
SCAS				
Mean (SD)	33.59^a^ (20.52)	15.51^a^ (9.47)	30.85 (19.83)	15.80 (10.20)
Range	6–77	3–44	6–76	3–44

IUS = Intolerance of Uncertainty scale (higher scores reflect greater intolerance of uncertainty); SSP = Short Sensory Profile (high scores reflect fewer sensory sensitivities); SCAS = Spence Children’s Anxiety scale (higher scores reflect greater anxiety)

^a^The mean SCAS score reported for our autistic sample is similar to that reported by Nauta et al. (2004) in a sample of 484 anxiety disordered children aged 6–11 years (Boys: M = 31.4, SD = 12.9; Girls: M = 33.0; SD = 13.5). Similarly, the mean SCAS score in our typical group is similar to those Nauta et al. report for their typical group (n = 261; Girls: M = 16.0, SD = 11.6; Boys: M = 15.9; SD = 9.0)

As expected, parents reported significantly greater levels of intolerance of uncertainty, *t*(113) = 11.19, *p* < .001, *d* = 1.89, sensory sensitivities, *t*(103) = 12.68, *p* < .001, *d* = 2.15, and anxiety, *t*(83) = 6.55, *p* < .001, *d* = 1.13, in autistic children compared to typical children. We also created subsamples of autistic (n = 40) and typical (n = 40) children, who were matched in terms of age, *t*(78) = .39, *p* = .70, *d* = .09 and full-scale IQ, *t*(78) = .16, *p* = .87, *d* = .04. Significant differences—with similar effect sizes—remained with regards to intolerance of uncertainty, *t*(78) = 8.21, *p* < .001, *d* = 1.84, sensory sensitivities, *t*(78) = 9.63, *p* < .001, *d* = 2.15, and anxiety, *t*(58) = 4.27, *p* < .001, *d* = .95, in the matched groups.^2^

Examination of the normative data on 1037 children provided by Dunn (1999) enabled us to classify these children in terms of the extent of their sensory atypicalities. Most of the autistic children (84 %) fell in the ‘definite difference’ range, with 8 % scoring in the ‘probable difference’ range and 8 % in the ‘typical performance’ range. By contrast, the majority (78 %) of typically developing children in this study fell into the ‘typical performance’ range (at or above 1 SD below the mean, where lower scores indicate greater levels of symptoms), with 14 % scoring in the ‘probable difference’ range and 8 % in the ‘definite difference’ range.

### Relationships with General and Developmental Variables

Next, we conducted Pearson correlation analyses to determine the relationship between scores on the three questionnaire measures (the Intolerance of Uncertainty scale, the Short Sensory Profile and Spence Children’s Anxiety scale) and children’s gender, age, ability (summed raw scores on the four subtests of the WASI-II, which were unadjusted for age), SCQ scores and ADOS-2 scores for each group separately (see Table 3). Because of the relatively large number of comparisons conducted, correlations are not reported as significant unless they reach a *p* value of at least .01. For autistic children, there were significant associations between their SCQ scores and Intolerance of Uncertainty scale scores, Short Sensory Profile scores and Spence Children’s Anxiety scale scores. Increased levels of autistic symptoms were associated with a greater degree of intolerance of uncertainty, sensory sensitivities and anxiety. There were no other significant associations (highest *r* = .23).

**Table 3:** Pearson’s correlations between scores on the Intolerance of Uncertainty scale, Short Sensory Profile and the Spence Children’s Anxiety scale and background variables (age, ability and autistic symptomatology), for children with autism (n = 64) and typically developing children (n = 85)

R	Gender	Age	Ability	SCQ	ADOS
IUS					
Autistic children	.05	.02	.19	.40*	.07
Typically developing children	.25	−.03	−.17		
SSP					
Autistic children	.05	.23	.01	−.37*	−.06
Typically developing children	−.02	.14	.18		
SCAS					
Autistic children	−.02	−.10	.01	.38*	−.01
Typically developing children	.18	−.18	−.16		

IUS = Intolerance of Uncertainty scale (higher scores reflect greater intolerance of uncertainty); SSP = Short Sensory Profile (high scores reflect fewer sensory sensitivities); SCAS = Spence Children’s Anxiety scale—Parent version (higher scores reflect greater anxiety); Ability = Summed raw scores on the four subtests of the Wechsler Abbreviated Scales of Intelligence, 2nd Edition; SCQ = Social Communication Questionnaire; ADOS = Autism Diagnostic Observation Schedule (n = 52). Higher scores on the SCQ and ADOS reflect greater autistic symptoms

* *p* < .01

### Correlations Between Intolerance of Uncertainty, Sensory Sensitivities and Anxiety

**Fig. 1:**
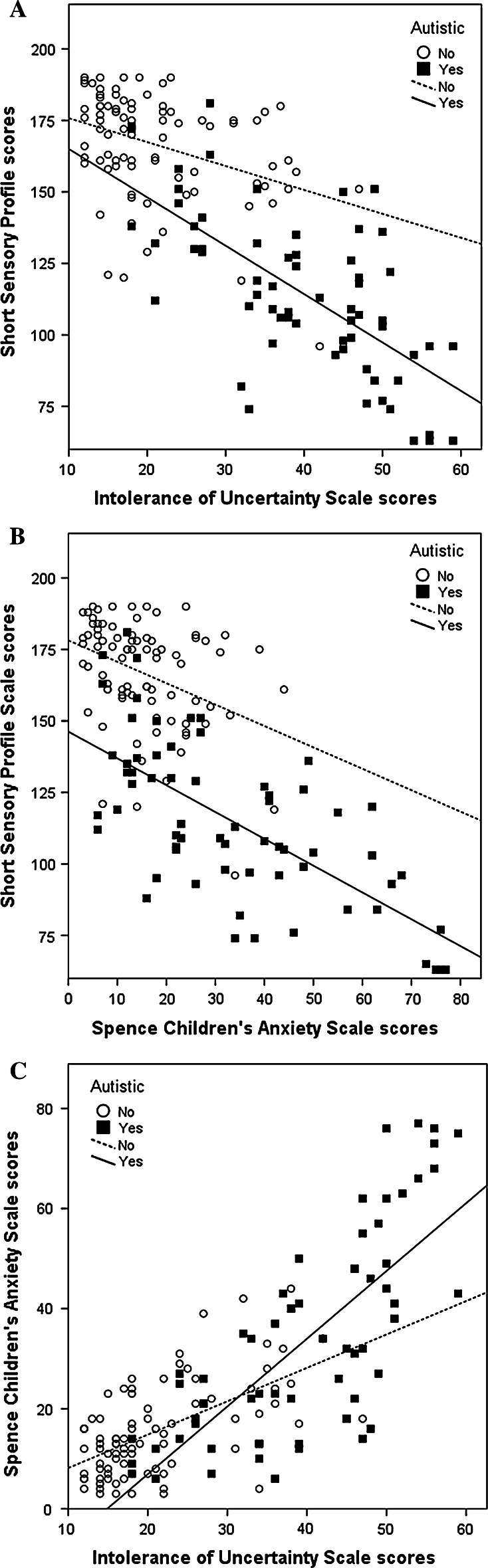
Scatterplots showing the relationship between parent reported Intolerance of Uncertainty scale scores and Short Sensory Profile scores (**a**), Spence Children’s Anxiety scale scores and Short Sensory Profile scores (**b**) and Intolerance of Uncertainty scale scores and Spence Children’s Anxiety scale scores (**c**) in both autistic and typically developing children. Higher scores on the Intolerance of Uncertainty scale and Spence Children’s Anxiety scale indicate greater levels of symptoms. Higher scores on the Short Sensory Profile indicate lower levels of symptoms

Correlational analyses were also used to determine the associations between scores on the three questionnaires for each group separately (see Table 4). There was a large, negative association (Cohen 1988) between scores on the Intolerance of Uncertainty scale and scores on the Short Sensory Profile in the autistic children, *r*(62) = −.67, *p* < .001, and a moderate association in the typical children, *r*(83) = −.37, *p* < .001. Similar correlations to those between the Intolerance of Uncertainty scale and the Short Sensory Profile were found between children’s scores on the Spence Children’s Anxiety scale and Short Sensory Profile [autistic: *r*(62) = −.68, *p* < .001; typical: *r*(83) = −.38, *p* < .001].^3^ Increased levels of intolerance of uncertainty *and* anxiety were associated with increased sensory sensitivities. As expected, there was also a strong positive association between scores on the Intolerance of Uncertainty scale and the Spence Children’s Anxiety scale in both the autistic, *r*(62) = .74, *p* < .001, and typical, *r*(83) = .59, *p* < .001, groups. Scatterplots showing the relationships between the three measures can be seen in Fig. 1.

**Table 4:** Pearson’s correlations between scores on the Intolerance of Uncertainty scale, Short Sensory Profile and Spence Children’s Anxiety scale for children with autism (n = 64) and typically developing children (n = 85)

	IUS-P	SSP	SCAS
IUS			
Children with autism	–		
Typical children	–		
SSP			
Children with autism	−.67*	–	
Typical children	−.37*	–	
SCAS			
Children with autism	.74*	−.68*	–
Typical children	.59*	−.38*	–

IUS = Intolerance of Uncertainty scale (parent report, higher scores reflect greater intolerance of uncertainty); SSP = Short Sensory Profile (high scores reflect fewer sensory sensitivities); SCAS = Spence Children’s Anxiety scale (parent report, higher scores reflect greater anxiety)

* *p* < .01

### Intolerance of Uncertainty and Anxiety as Predictors of Children’s Sensory Sensitivities

To address our second and third aims, we first conducted hierarchical regression analysis to determine the unique contribution of intolerance of uncertainty and anxiety to children’s sensory sensitivities, as measured by their Short Sensory Profile scores, for each group separately (see Table 5). Age, IQ and gender were not significantly associated with children’s sensory sensitivities (see above); these variables were therefore not entered as covariates in the analysis. Scores on the Intolerance of Uncertainty scale were entered in the first step, followed by scores on the Spence Children’s Anxiety scale in the second step. For autistic children, Intolerance of Uncertainty scores made a significant contribution to the model [R^2^ change = .45, *F*(1, 62) = 50.01, *p* < .001]. Spence Children’s Anxiety scale scores made a small additional contribution to the variance in children’s sensory sensitivities [R^2^ change = .08, *F*(1, 61) = 9.63, *p* < .001]. The final model [*F*(2, 61) = 33.3, *p* < .001] explained 52 % of the variance in children’s Short Sensory Profile scores.

**Table 5:** Intolerance of Uncertainty scale scores and Spence Children’s Anxiety scale scores as predictors of Short Sensory Profile scores in children with autism and typically developing children

	*R* ^2^	∆*R* ^2^	∆*F*	β
Children with autism (n = 64)				
Model 1	.45	.45	50.01*	
IUS				−.67*
Model 2	.52	.08	9.63*	
IUS				−.37*
SCAS				−.41*
Typically developing children (n = 85)				
Model 1	.14	.14	13.49*	
IUS				−.37*
Model 2	.18	.04	3.76	
IUS				−.23
SCAS				−.24

IUS-P = Intolerance of Uncertainty scale (higher scores reflect greater intolerance of uncertainty); SCAS = Spence Children’s Anxiety scale (higher scores reflect greater anxiety)

* *p* < .01

**Fig. 2:**
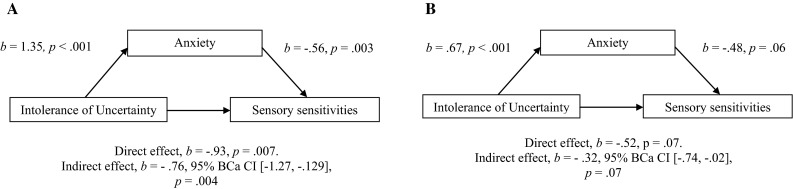
Models of intolerance of uncertainty as a predictor of sensory sensitivities, mediated by anxiety, for children with autism (**a**) and typically developing children (**b**). The confidence intervals for the indirect effect is a BCa bootstrapped CI based on 1000 samples

As shown in Table 5, children’s Intolerance of Uncertainty scale scores remained a significant (albeit somewhat weaker) predictor of their Short Sensory Profile scores, even after Spence Children’s Anxiety scale scores were added to the model. This finding suggests that the relationship between intolerance of uncertainty and sensory sensitivities is partly but not fully mediated by anxiety. We therefore performed mediation analysis to assess the size of the indirect effect (see Fig. 2a). This analysis and the ones performed below were based on the PROCESS tools for moderation and mediation analysis (Hayes 2012). Significant relationships in the models are indicated by bias-corrected and accelerated bootstrapped confidence intervals (based on 1000 samples) that do not overlap with zero.

The mediation analysis confirmed that for children with autism, the relationship between Intolerance of Uncertainty scale scores and Short Sensory Profile scores was mediated by Spence Children’s Anxiety scale scores (*b* = −.76, BCa CI [−1.27, −.29], *p* = .004) and that the size of this indirect effect was large (*κ*^2^ = .27, 95 % BCa CI [.11, .41]). As expected, the direct effect of Intolerance of Uncertainty scale scores on Short Sensory Profile scores was also significant (*b* = −.93, *p* = .007). These results indicate that anxiety can only partly explain the relationship between intolerance of uncertainty and sensory sensitivities. Intolerance of uncertainty and sensory sensitivities are also associated with each other, independently of anxiety.

Table 5 suggests that there are key differences between the autistic and typical groups in the magnitude of the relationships between measures. When typical children’s Intolerance of Uncertainty scores were entered in step 1, they made a significant contribution to the model [R^2^ change = .14, *F*(1, 83) = 13.48, *p* < .001]. Unlike autistic children, however, Spence Children’s Anxiety scale scores failed to make a significant and unique contribution to typical children’s Short Sensory Profile scores over and above Intolerance of Uncertainty scores [R^2^ change = .04, *F*(1, 82) = 3.74, *p* = .057]. The final model [*F*(2, 82) = 8.83, *p* < .001] explained 17.7 % of the variance in children’s Short Sensory Profile scores. In a mediation analysis (see Fig. 2b), the Sobel test for an indirect effect of Intolerance of Uncertainty scale scores on Sensory Profile scores through Spence Children’s Anxiety scale scores did not reach significance (*b* = −.32, *p* = .07). Nevertheless, the more reliable bootstrapped confidence intervals for the size of the indirect effect did not contain zero, BCa CI [−.74, −.02], indicating that mediation had indeed taken place and that the non-significant effect was most likely due to a lack of power. The strength of the indirect effect was medium (*κ*^2^ = .12, BCa CI [.01, .25]). These results suggest that the relationship between intolerance of uncertainty and sensory sensitivities in typical children can also partly be explained by anxiety, but to a lesser extent than in autistic children. There was not a significant direct effect of Intolerance of Uncertainty on Short Sensory Profile scores either (*b* = −.52, CI [−1.07, .03], *p* = .07) but the total effect, which is a combination of the direct and indirect effects, was significant (*b* = −.84, *p* < .001).

### Intolerance of Uncertainty as a Mediator Between Autism and Anxiety and Autism and Sensory Sensitivities

**Fig. 3:**
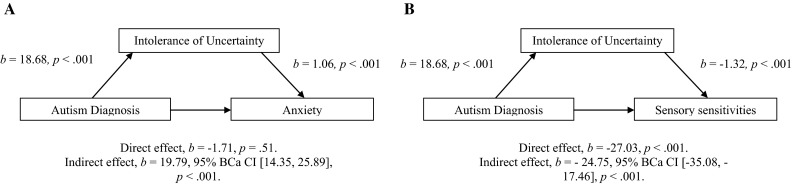
Models of autism diagnosis as a predictor of anxiety, mediated by intolerance of uncertainty (**a**) and as a predictor of sensory sensitivities, mediated by intolerance of uncertainty (**b**). The confidence intervals for the indirect effect is a BCa bootstrapped CI based on 1000 samples

The final aim of our study was to compare the role of intolerance of uncertainty as a potential mediator in the relationship between autism and anxiety, to its role as a mediator in the relationship between autism and sensory sensitivities. The findings of Boulter et al. (2014) indicated that intolerance of uncertainty mediated the relationship between autism and anxiety. After controlling for levels of intolerance of uncertainty, they found that having an autism diagnosis was no longer associated with higher levels of anxiety. We attempted to replicate this finding using a mediation model (see Fig. 3a). Autism diagnosis, entered as a binary variable, had a significant, and large, indirect effect on Spence Children’s Anxiety scale scores through Intolerance of Uncertainty scale scores (*b* = 19.79, BCa CI [14.35, 25.89], *κ*^2^ = .50), but not a significant direct effect (*b* = −1.71, CI [−6.82, 3.40), *p* = .51).^4^

Next, we extended this analysis to investigate whether autism diagnosis would still predict sensory sensitivities when intolerance of uncertainty scores were taken into account. Again, using a mediation model (see Fig. 3b) we found a significant indirect effect of autism diagnosis on Short Sensory Profile scores through Intolerance of Uncertainty scores (*b* = −24.75, BCa CI [−35.08, −17.46], *p* < .001, *κ*^2^ = .38), but also a direct effect of a similar size (*b* = −27.03, CI [−35.91, −18.16], *p* < .001). Adding Spence Children’s Anxiety scale scores in as an additional mediator did not reduce the size of the direct effect of autism diagnosis on Short Sensory Profile scores (*b* = −28.06, CI [−36.43, −19.68], *p* < .001). These results suggest that intolerance of uncertainty has greater explanatory power in the relationship between autism and anxiety than between autism and sensory sensitivities, but nevertheless has an important mediating role in the latter relationship.

## Discussion

This study investigated the relationship between intolerance of uncertainty and sensory sensitivities in groups of autistic and typical children. Consistent with previous research (Boulter et al. 2014; Chamberlain et al. 2013; Tomchek and Dunn 2007), parents of children on the autism spectrum reported greater levels of intolerance of uncertainty, anxiety and sensory sensitivities in their children than parents of typically developing children. Critically, we extended previous research by showing that intolerance of uncertainty explained approximately half the variance in autistic children’s sensory sensitivity scores and this relationship was partially mediated by children’s anxiety levels. Although the nature of the relationship was similar for both groups, intolerance of uncertainty explained considerably less of the variance (one third, in fact) in typically developing children’s sensory sensitivities. In addition, although anxiety scores partly explained the relationship between intolerance of uncertainty and sensory sensitivities in children with autism, intolerance of uncertainty and sensory sensitivities remained significantly associated in this group even after the effects of anxiety were controlled for.

The significant positive association between intolerance of uncertainty, anxiety and sensory sensitivities in children with autism replicates Wigham et al.’s (2015) findings, and extends it further through the inclusion of a comparison group of typically developing children. There are a number of possible explanations for the stronger correlation between intolerance of uncertainty in the autistic group (*r* = −.67) compared to the typical group (*r* = −.38). First, although there was a wide range of scores in both groups on all measures (see Table 2), with some parents even reporting sensory atypicalities in their typical children, a lack of variation in scores may have precluded the possibility of observing a stronger relationship between sensory sensitivities and intolerance of uncertainty in the typical group. Second, the processes at work might be the same in children with and without autism, but the relationship between intolerance of uncertainty and sensory sensitivities could be stronger when individuals have higher scores on both measures. Future research which investigates the levels of sensory sensitivities in individuals who have high levels of intolerance of uncertainty and anxiety but who are not autistic would be informative here.

Third, the scale used to measure intolerance of uncertainty may have tapped a distinct underlying process in the group of autistic children, regarding the way individuals deal with uncertainty. The current study was guided by a theoretical account of autistic perception put forward by Pellicano and Burr (2012), who hypothesised that difficulties utilising prior experience when processing inherently ambiguous sensory information give rise to a greater reliance on bottom-up sensory signals and, subsequently, differences in the way that autistic individuals interpret sensory information. The finding that intolerance of uncertainty had a direct effect on autistic children’s sensory sensitivities, over and above anxiety, supports this explanation.

In addition to explaining the direct effect of intolerance of uncertainty on autistic children’s sensory sensitivities, it may be possible to extend Pellicano and Burr (2012) to include the mediating role of anxiety found here. Difficulties generating predictions at the computational or neural level may translate to beliefs at the psychological level that uncertainty is negative and potentially threatening. This ‘intolerance of uncertainty’ may in turn engender attempts to decrease uncertainty; manifesting in anxiety symptoms such as rumination about the possibility of various negative outcomes and an attentional bias to potentially threatening stimuli in the environment. In this hypervigilant state, individuals may be more likely to notice and respond to aversive sensory stimuli, and less likely to successfully disengage from potentially threatening sensory stimuli (Green and Ben-Sasson 2010).

Although we have a priori theoretical reasons (e.g., Pellicano and Burr 2012) to support this particular causal story, we must be cautious about doing so given the cross-sectional nature of the data. It is also plausible that sensory sensitivities might *cause* anxiety and intolerance of uncertainty. Donna Williams, who is autistic, describes how “sensory overload caused by bright lights, fluorescent lights, colours, and patterns makes the body react as if being attacked or bombarded, resulting in such physical symptoms as headaches, anxiety, panic attacks or aggression” (Williams 1994, p. 43). Consistent with this view, there is evidence to suggest that sensory over-responsivity predicts the development of anxiety in toddlers (Green et al. 2012). Experimental intervention studies would be helpful in elucidating the causal or bidirectional relationships between the full range of sensory sensitivities, anxiety and intolerance of uncertainty, and the mechanisms which underpin them.

Future research in this area would also benefit from the collection of self-report data from children with and without autism in regards to their own sensory sensitivities, in addition to their levels of intolerance of uncertainty and anxiety. There is some evidence to suggest that the differences in anxiety levels between autistic and typically developing children on the Spence Children’s Anxiety scale are more pronounced among parent-reported, than child-reported data (Boulter et al. 2014), but whether this discrepancy is replicated in reports of sensory sensitivities is as yet unknown. Using varied methods of measurement, such as both self- and other-report, would also reduce potential bias due to common method variance.

In conclusion, intolerance of uncertainty is well established as an important construct in the development of affective symptoms (Calleo et al. 2010; Dugas et al. 2012; Whiting et al. 2014). Our study shows that it also appears to be highly relevant to sensory sensitivities, particularly among autistic children. Sensory interventions in autism are common (Green et al. 2006) yet there is huge variation in the principles underlying these interventions, the behaviours they target, and the methodologies that are applied, as well as a lack of rigorous evaluation of their effectiveness (see review by Case-Smith et al. 2015). Further investigation into the relationships between these constructs could be vital for the development of effective interventions for sensory sensitivities in children with and without autism.

## Footnotes

1 The term ‘autistic person’ is the preferred language of many people on the spectrum (see Sinclair 1999, and Kenny et al. 2015). In this article, we use this term as well as person-first language to respect the wishes of all individuals on the spectrum.

2 Equal variances were not assumed. We therefore report the degrees of freedom and accompanying statistics adjusted accordingly.

3 In keeping with Wigham et al. (2015), scores on the SSP minus items assessing the underresponsiveness/seeks sensation, auditory filtering and low energy/weak subscales were also calculated to create ‘sensory over responsiveness’ scores. These scores had a similar correlation with IUS scores to total scores for both autistic (*r* = −.68) and typically developing children (*r* = −.38). These scores also had a similar correlation with SCAS scores to total scores for both children with autism (*r* = −.70) and typically developing children (*r* = −.38).

4 When we performed the analysis with anxiety as the mediating variable, we found that anxiety did significantly mediate the autism—intolerance of uncertainty relationship, but the size of the indirect effect was smaller when anxiety was positioned as a mediating variable (indirect effect, *b* = 7.78, 95 % BCa CI [5.36, 10.28], *p* < .001, *κ*^2^ = .35) than when intolerance of uncertainty was positioned as a mediating variable (indirect effect, *b* = 19.79, 95 % BCa CI [14.35, 25.89], *p* < .001, *κ*^2^ = .50). These results provide further support for the theoretical position we adopt here.
